# CUEDC2, a novel interacting partner of the SOCS1 protein, plays important roles in the leukaemogenesis of acute myeloid leukaemia

**DOI:** 10.1038/s41419-018-0812-6

**Published:** 2018-07-10

**Authors:** Qing-Yun Wu, Yuan-Yuan Zhu, Yang Liu, Fang Wei, Yu-Xue Tong, Jiang Cao, Ping Zhou, Ming-Shan Niu, Zhen-Yu Li, Ling-Yu Zeng, Feng Li, Kai-Lin Xu

**Affiliations:** 1 Blood Diseases Institute, Xuzhou Medical University, Xuzhou, Jiangsu China; 2 Department of Hematology, the Affiliated Hospital of Xuzhou Medical University, Xuzhou, Jiangsu China; 3 Department of Cell Biology and Neurobiology, Xuzhou Key Laboratory of Neurobiology, Xuzhou Medical University, Xuzhou, Jiangsu 221002 China

## Abstract

Downregulation of suppressor of cytokine signalling-1 (SOCS1) is one of the vital reasons for JAK1-STAT3 pathway activation in acute myeloid leukaemia (AML). CUE domain-containing 2 (CUEDC2) was a novel interacting partner of SOCS1 and a positive correlation between the expression of CUEDC2 and SOCS1 was confirmed in primary AML cells and AML cell lines without SOCS1 promoter methylation. We aimed to explore roles of CUEDC2 in regulating ubiquitin-mediated degradation of SOCS1 in the leukaemogenesis of AML.

According to in vitro experiments, CUEDC2 overexpression increased the level of SOCS1 protein, suppressed JAK1-STAT3 pathway activation. The suppression of this pathway inhibited AML cells’ proliferation by causing G1 arrest and enhanced AML cells’ sensitivity to cytarabine and idarubicin. Similarity, downregulation of CUEDC2 produced opposite results. Knockout or low expression of CUEDC2 in mouse or AML patients displayed lower overall survival and event-free survival rates, compared with these mouse and AML patients had high-CUEDC2 expression. Mechanistic studies revealed that CUEDC2 overexpression attenuated SOCS1 ubiquitination, facilitated its stabilisation by enhancing SOCS1, Elongin C and Cullin-2 (CUL2) interactions, thus inhibited JAK1-STAT3 pathway and leukaemogenesis of AML. Therefore, our novel findings indicated that CUEDC2 interacted with SOCS1 to suppress SOCS1’s ubiquitin-mediated degradation, JAK1-STAT3 pathway activation and leukaemogenesis of AML.

## Introduction

Despite of the improved outcomes of acute myeloid leukaemia (AML) in recent years, most patients will suffer relapse receiving chemotherapy alone. Deep explore of the molecular mechanism of AML is very important for translational research to improve the survival of patients. The hyperactivation of JAK1-STAT3 pathway plays vital roles in leukaemogenesis and relapse of AML^1,2^. The inhibition of JAK1-STAT3 pathway represents a promising therapeutic strategy for AML patients. Many JAK1-STAT3 pathway inhibitors have been developed based on its known activation mechanism. However, the efficacy was not confirmed in recent clinical trials^3,4^. Thus, other mechanisms underlying JAK1-STAT3 signalling hyperactivation in AML need to investigate.

The suppressors of cytokine signalling (SOCS) proteins are important for regulating of JAK-STAT pathway^5^. More importantly, downregulation of SOCS1 is a key reason for JAK1-STAT3 pathway activation and leukaemogenesis of AML^6,7^. SOCS1 negatively regulates JAK1-STAT3 pathway through three mechanisms. First, SOCS1 binds to the activation loop of JAK1 via its SH2 domain and inhibits JAK1’s kinase activity^8^. Second, SOCS1 regulates the activity of this pathway by SOCS box-mediated proteasomal degradation of JAKs^9^. Third, SOCS1 binds to the phospho-tyrosine residues on the receptors and physically blocks STATs from binding to their receptors^9,10^. Hypermethylation of SOCS1 promoter and elevated ubiquitin-mediated degradation were main mechanisms of SOCS1 downregulation in AML^11,12^. The mechanism of SOCS1 promoter hypermethylation has been intensively studied and almost completely clarified. Although the Eongin BC complex, which interacts with the SOCS box, has been shown to increase the SOCS1 content by inhibiting its degradation^13^, the mechanism how SOCS1 degradation is regulated in AML remains unclear. Thus, studies aiming to elucidate which gene or protein might be involved in regulating SOCS1’s ubiquitin-mediated degradation and its degradation regulating mechanism in AML are of great importance.

The CUE domain-containing protein 2 (CUEDC2), a novel interacting partner and a potential regulator of the ubiquitin-mediated degradation of SOCS1, is a promising target of treatment. CUEDC2 plays key roles in protein ubiquitin-mediated degradation^14^, inflammation, tumour development^15^, and chromosomal instability^16^. Identified as ubiquitin-binding motifs, CUE domains interact with both mono and polyubiquitin and play dual roles in recognising mono and polyubiquitin as well as in facilitating intramolecular monoubiquitination^14,17^. CUEDC2 might be a novel regulator of SOCS1’s ubiquitin-mediated degradation and an inhibitor of the JAK1-STAT3 pathway. However, whether CUEDC2 was involved in regulating SOCS1’s ubiquitin-mediated degradation and the leukaemogenesis of AML remains unclear.

In this study, we found that CUEDC2 overexpression attenuated SOCS1 ubiquitination, facilitated its stabilisation by enhancing SOCS1, Elongin C and cullin-2 (CUL2) interactions, thus inhibited JAK1-STAT3 pathway and leukaemogenesis of AML. Therefore, our novel findings indicated that CUEDC2 interacted with SOCS1 to suppress SOCS1’s ubiquitin-mediated degradation, JAK1-STAT3 pathway activation and leukaemogenesis of AML.

## Results

### SOCS1 expression was downregulated in primary AML cells and AML cell lines

**Fig. 1:**
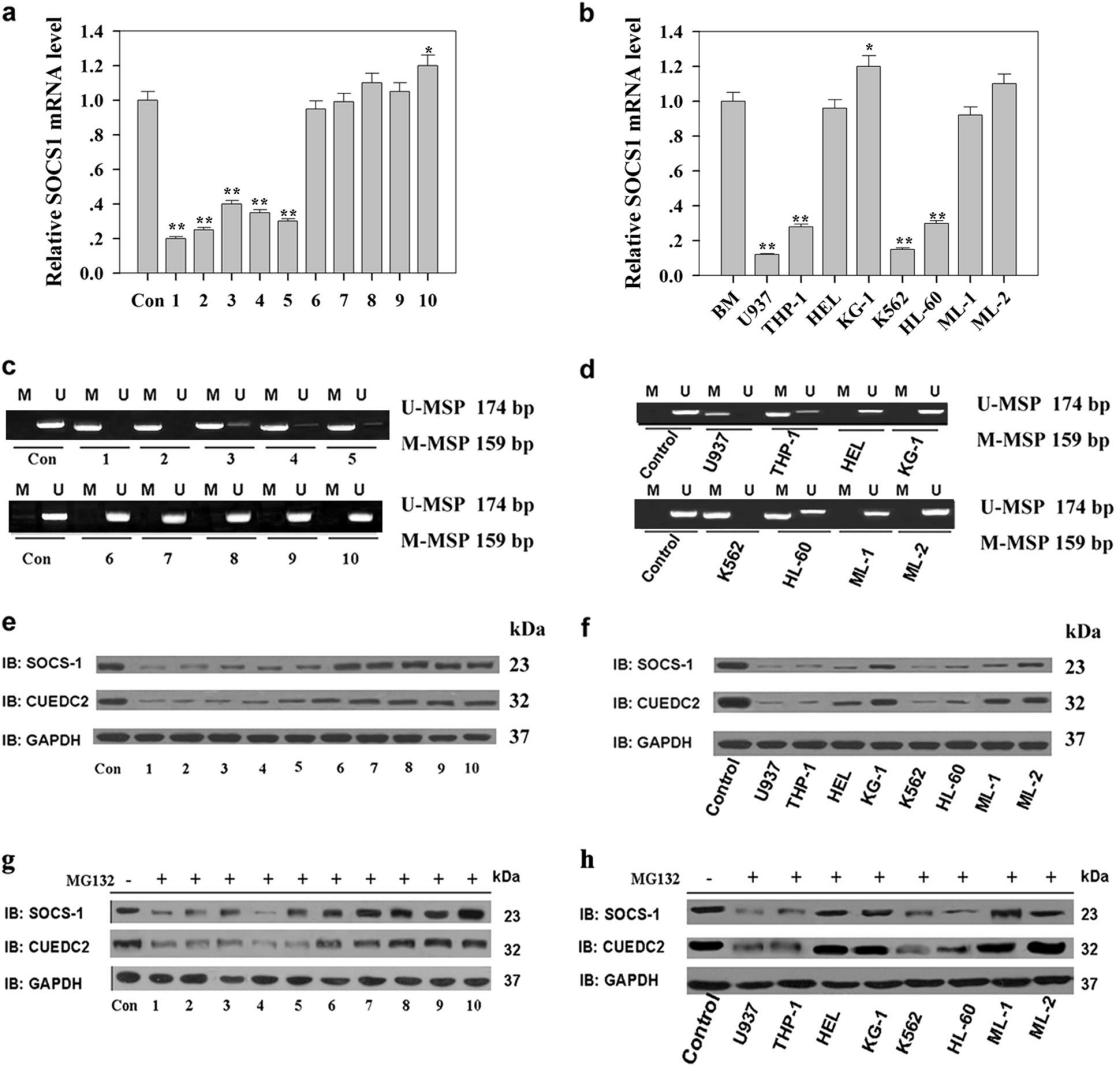
The downregulation of SOCS1 observed in the primary AML cells and AML cell lines was mainly caused by the hypermethylation of its promoter and the elevated ubiquitin-mediated degradation. **a** and **b** Levels of the SOCS1 mRNA in primary AML cells and AML cell lines were detected by quantitative real-time PCR. Ten primary AML cells were initially sorted with magnetic beads, and then the total RNA was isolated and the SOCS1 mRNA levels were detected by RT-PCR; bone marrow cells from the healthy donors were used as a control. **c** and **d** The methylation of the SOCS1 promoter in primary AML cells and AML cell lines was detected by methylation-specific polymerase chain reaction (MSP). The MSP for promoter methylation was performed, as described in a previous study^18^. MSP primers were designed to amplify the methylated (M-MSP) and unmethylated (U-MSP) alleles, and the size of products is indicated in the figure. **e** and **f** The expression of SOCS1 and CUEDC2 in primary AML cells and cell lines was detected by western blotting. **g** and **h** The expression of SOCS1 and CUEDC2 in the same primary AML cells and AML cell lines used in Fig. 1e, f were treated with MG132 for 6 h and then detected by western blotting. The mononuclear cells were initially sorted on a Ficoll gradient after isolation from the bone marrow of AML patients and then cultured and treated with MG132 in the OP-9-containing flask. After 6 h, cells were harvested and the expression of SOCS1 and CUEDC2 was detected by western blotting. In all experiments, the normal bone marrow cells from healthy volunteers were used as a control, and the experiments were conducted at least three times. *P *<* 0.05, **P *<* 0.01

The expression and methylation of SOCS1’s promoter in primary AML cells and AML cell lines were detected to analyse mechanisms underlying its downregulation. In approximately 48.4% of primary AML cells and 50% of AML cell lines, the mRNA level of SOCS1 was lower (Fig. 1a, b) and its promoter methylation was higher (Fig. 1c, d) than that in bone marrow cells from healthy donors. Thus, low-SOCS1 expression in these AML cells was caused by SOCS1 promoter hypermethylation. In other approximately 46.5% of primary AML cells and 50% of AML cell lines, the mRNA level of SOCS1 (Fig. 1a, b) was similar to that observed in bone marrow cells from healthy donors, and the SOCS1 promoter methylation was not observed. However, the level of SOCS1 protein in these cells was lower than that in bone marrow cells from healthy donors (Fig. 1e, f). Thus, the low-SOCS1 expression observed in these portions of AML cells was regulated at the posttranscriptional level (Fig. 1a–f).

In order to determine whether SOCS1 expression in these AML cells without SOCS1 promoter methylation was regulated by ubiquitin-mediated degradation, the same AML cells and samples used in SOCS1 protein levels detected (Fig. 1e, f) were treated with MG132. As is shown in Fig. 1, the SOCS1 protein levels in these AML cells without SOCS1 promoter methylation after MG132 treatment (Fig. 1g, h) was higher than that in these AML cells before MG132 treatment (Fig. 1e, f). Thus, the low-SOCS1 expression observed in these AML cells was regulated by ubiquitin-mediated degradation. Consistent with previous studies^18^, this study also suggested that downregulation of SOCS1 in AML cells was caused by SOCS1 promoter methylation and ubiquitin-mediated degradation.

After exploration downregulation mechanisms of SOCS1 in AML, correlation between SOCS1 expression and its promoter methylation to AML subtypes and lineages of primary AML cells were analysed. The SOCS1 expression reduction was most pronounced in U937 and THP-1 monocytic cell lines among all analysed AML cell lines, followed by K562 and HL-60 APL cell line, the human erythroleukemia cell (HEL) and KG-1 erythroleukaemia cell lines, and ML-1 and ML-2 myelomonocytic cell lines (Fig. 1). Similarity, the methylation of SOCS1 promoter was mainly observed in monocytic cell lines U937 and THP-1, K562 and the HL-60. However, the SOCS1 promoter methylation was not detectable in erythroleukaemia cell lines HEL and KG-1 or in myelomonocytic cell lines ML-1 and ML-2. Furthermore, information about lineages of primary AML cells exhibiting reduced SOCS1 expression was also summarised, our results indicated that inv(16)/t(16;16) AML (*n* = 56) displayed the most obvious reduction in SOCS1 levels among all detected primary AML cell subtypes, followed by *t*(8;21) AML (*n* = 63), *t*(11q23)/MLL AML (*n* = 34), *t*(15;17) AML (*n* = 77) and complex AML (*n* = 45) (data not shown). Similarly, correlations between SOCS1 expression and its promoter methylation with primary AML cells subtypes were also analysed; leukaemia with normal karyotype displayed the highest level of SOCS1 promoter methylation (87.07%, *n* = 116) among all detected primary AML cells subtypes, followed by *t*(15;17) AML (80.52%, *n* = 77), complex AML (80%, *n* = 45), *t*(11q23)/MLL AML (38.23%, *n* = 34), t(8;21) AML (23.81%, *n* = 63) and inv(16)/*t*(16;16) AML (14.29%, *n* = 56) (data not shown). Thus, our result indicated that the SOCS1 expression and its promoter methylation had a closely relationship to AML subtypes and lineages of primary AML cells.

### CUEDC2, a novel interacting protein and potential regulator of the ubiquitin-mediated degradation of SOCS1, interacted with the SH2 domain of SOCS1 via its CUE domain

**Fig. 2:**
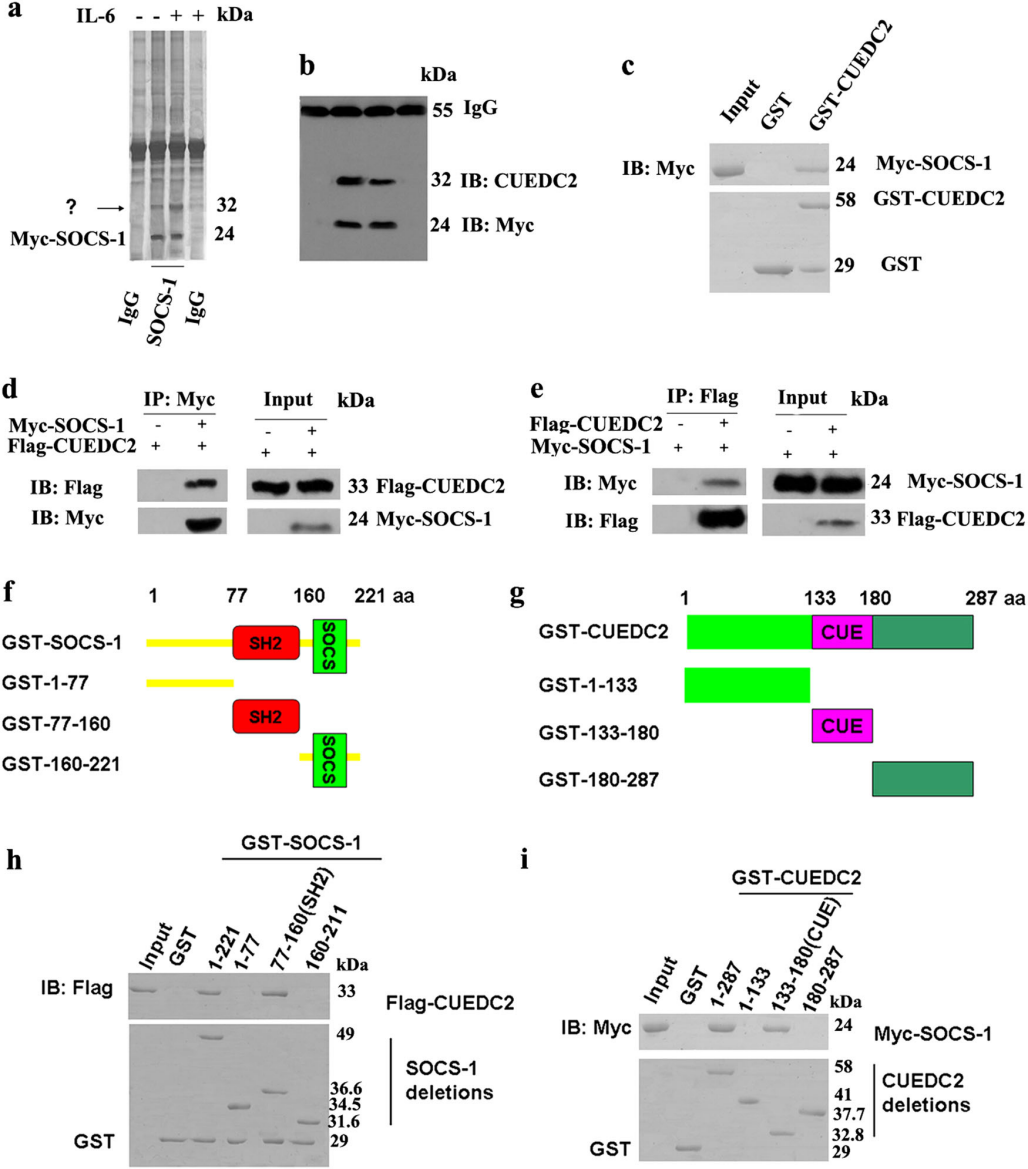
CUEDC2 interacted with the SH2 domain of SOCS1 via its CUE domain. **a** Myc-SOCS1 was overexpressed in the KG-1 cell line, and all possible interaction proteins were obtained by immunoprecipitation using a SOCS1-specific antibody; protein bands were then detected by silver staining. **b** Western blot analysis of the levels of the CUEDC2 and Myc proteins using specific antibodies and lysates from KG-1 cells overexpressing Myc-SOCS1. **c** Purified GST or GST-CUEDC2 fusion proteins were incubated with cell lysates from the KG-1 cells transfected with the Myc-SOCS1 vector. After extensive washes, bound proteins were analysed by immunoblotting (IB) with an anti-Myc antibody. The GST fusion proteins were resolved by SDS-PAGE and stained with Coomassie Blue. **d** KG-1 cells were transfected with Flag-CUEDC2 and Myc-SOCS1 expression vectors, as indicated. Whole cell lysates were then immunoprecipitated (IP) with an anti-Myc monoclonal antibody and immunoblotted with an anti-Flag antibody. **e** KG-1 cells were transfected with Flag-CUEDC2 and Myc-SOCS1 expression vectors, as indicated. Whole cell lysates were then immunoprecipitated with an anti-Flag monoclonal antibody and immunoblotted with an anti-Myc antibody. **f** and **g** Schematics of the series of GST-SOCS1 and GST-CUEDC2 deletion mutations used in domain-mapping experiments; numbers indicate the amino acids included in the constructs. **h** Purified GST or truncated GST-SOCS1 fusion proteins were incubated with lysates from the KG-1 cells that had been transiently transfected with the Flag-CUEDC2 vector. After extensive washes, bound proteins were analysed by immunoblotting with an anti-Flag antibody. GST fusion proteins were resolved by SDS-PAGE and stained with Coomassie Blue. **i** Purified GST or truncated GST-CUEDC2 fusion proteins were incubated with lysates from the KG-1 cells that had been transiently transfected with the Myc-SOCS1 vector. After extensive washes, bound proteins were analysed by immunoblotting with an anti-Myc antibody. GST fusion proteins were resolved by SDS-PAGE and stained with Coomassie Blue

Mass spectrometry (MS) was used to investigate specific proteins that might be involved in regulating the ubiquitin-mediated degradation of SOCS1. Using a shotgun analysis, 289 proteins were identified, and only 87 proteins that might actually interact with SOCS1 are shown (Table S1), as at least 4 unique peptides were detected for these proteins. Moreover, approximately 21 proteins have been proved to interact with SOCS1 was also detected, indicating that results from our detection method were believable (Table S1). Interestingly, many proteins that have been reported to play vital roles in SOCS1 degradation were also detected. Most importantly, a novel protein that interacted with SOCS1, CUEDC2 was identified as potentially involved in regulating the ubiquitin-mediated degradation of SOCS1. In the presence of overexpressed SOCS1, the expression of the 32 kDa band of CUEDC2 was increased (Fig. 2a), which was verified by immunoblotting using a CUEDC2-specific antibody (Fig. 2b). Moreover, GST-pulldown experiments showed that Myc-SOCS1 was pulled down by GST-CUEDC2, but not by GST alone (Fig. 2c). Immunoprecipitation experiments indicated that the Flag-CUEDC2 was immunoprecipitated by a Myc-specific antibody (Fig. 2d). Similarly, Myc-SOCS1 was immunoprecipitated by a Flag-specific antibody (Fig. 2e). These results implied that CUEDC2 interacted with SOCS1.

In order to determine interaction domains between SOCS1 and CUEDC2, GST-pulldown experiments were done. A series of GST-SOCS1 and GST-CUEDC2 deletion mutations were constructed (Fig. 2f, g). As indicated in Fig. 2h, CUEDC2 was only pulled down by the full-length SOCS1 protein and its SH2 domain. Similarly, SOCS1 was only pulled down by the full-length CUEDC2 protein and its CUE domain (Fig. 2i). Therefore, SOCS1 interacted with the CUE domain of CUEDC2 via its SH2 domain.

Since CUEDC2 was involved in protein ubiquitin-mediated degradation and interacted with SOCS1, the correlation between SOCS1 and CUEDC2 in AML cells without SOCS1 promoter methylation was analysed. Pearson’s correlation coefficients were calculated to determine the correlation between the decreased levels of CUEDC2 protein (compared to those in cells from healthy donors) and the decreased levels of SOCS1 protein in primary AML cells and AML cell lines without SOCS1 promoter methylation. Our result indicated that the coefficient of correlation between decreased protein levels of CUEDC2 and decreased protein levels of SOCS1 in primary AML cells without SOCS1 promoter methylation was >0.9 (Table S2). Combined with results from Fig. 1, one can deduce that low CUEDC2 expression was a key reason for ubiquitin-mediated degradation of SOCS1 in AML cells without SOCS1 promoter methylation, and CUEDC2 was a potential regulator of the ubiquitin-mediated degradation of SOCS1.

### CUEDC2 inhibited the JAK1-STAT3 signalling pathway

**Fig. 3:**
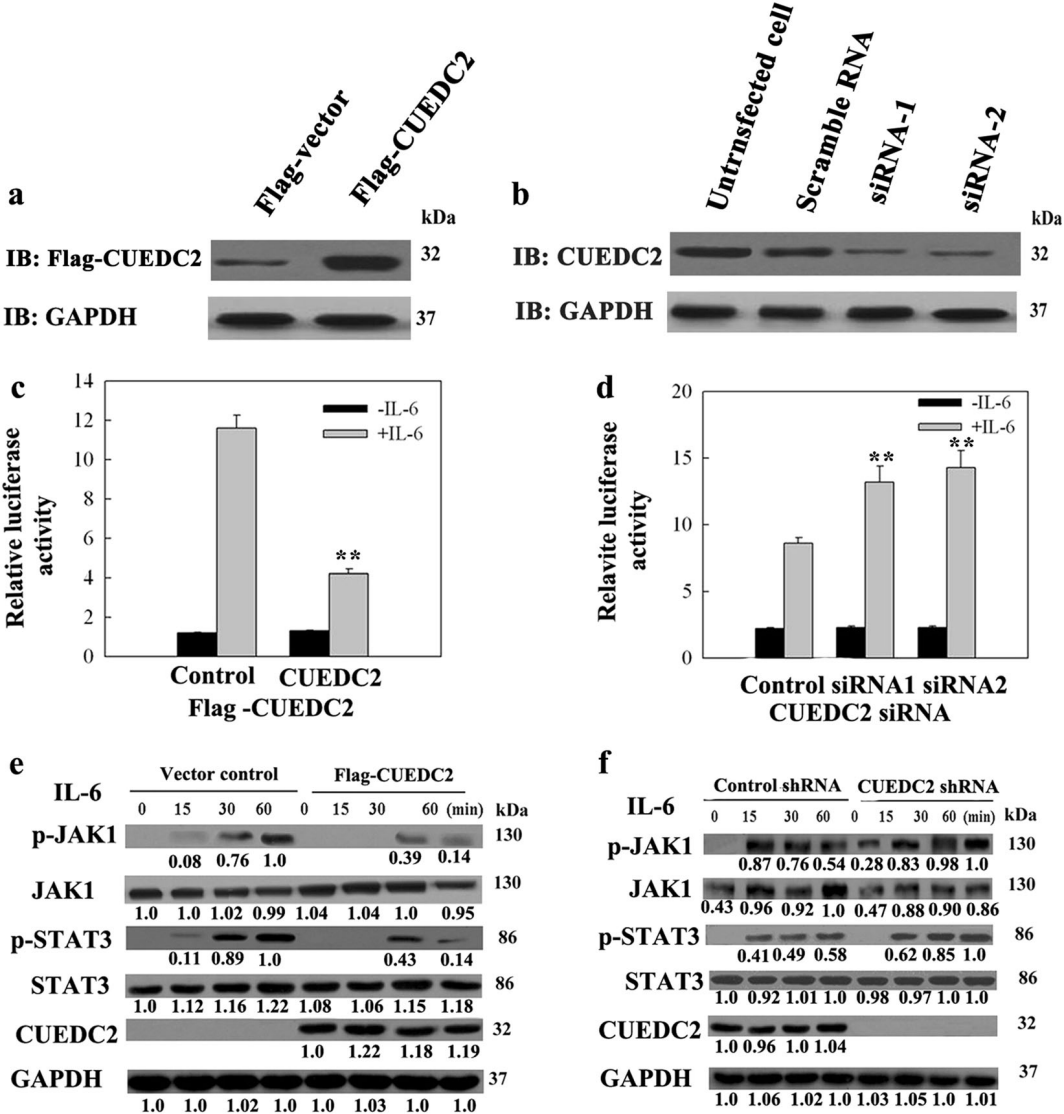
CUEDC2 overexpression inhibited the activation of the JAK1-STAT3 pathway, whereas CUEDC2 knockdown promoted the activation of the JAK1-STAT3 pathway. **a** HEL cells were transfected with the Flag vector or Flag-CUEDC2 vector by electroporation. after transfection, the efficiency of CUEDC2 overexpression was determined by immunoblotting with an anti-Flag antibody. **b** KG-1 cells were transfected with a control siRNA (scrambled RNA) or CUEDC2 siRNA by electroporation; 48 h after transfection, the efficiency of CUEDC2 knockdown was determined by immunoblotting with a CUEDC2 antibody. **c** HEL cells were transfected with the pACT luciferase reporter, HA-STAT3 and Flag-CUEDC2 vectors by electroporation. Forty-eight hour after transfection, cells were treated with IL-6 for 6 h and luciferase activity was measured. Renilla reporter pRL-TK vectors were used as an internal control of transfection efficiency. **P *<* 0.01. **d** KG-1 cells were cotransfected with a scrambled RNA or CUEDC2 siRNA, pACT-Luc and HA-STAT3 plasmids by electroporation, and after 48 h, cells were treated with IL-6 for 6 h and luciferase reporter assays were performed. **P *<* 0.01. **e** HEL cells overexpressing CUEDC2 were seeded in six-well plates, stimulated with IL-6 for the indicated periods, and total cell lysates were immunoblotted using JAK1, pJAK1, STAT3, pSTAT3, CUEDC2 and GAPDH antibodies. **f** KG-1 cells in which CUEDC2 was stably knocked down or transfected with the scrambled RNA were seeded in six-well plates, treated with IL-6 for the indicated periods, and then an immunoblot analysis was performed. The bands shown in Fig. 3e, f were quantified using ImageJ software. The highest levels of JAK1, pJAK1 and pSTAT3 were set 1.0, whereas the first bands for pSTAT3, CUEDC2 and GAPDH that appeared in Fig. 3e, f were set to 1.0

A luciferase reporter assay was performed to elucidate whether CUEDC2 affected the JAK1-STAT3 pathway. HEL and KG-1 cell lines were cotransfected with the pACT-Luc luciferase reporter plasmid, HA-STAT3 and Flag-CUEDC2 or the CUEDC2 siRNA, respectively. Since CUEDC2 regulated SOCS1 expression at posttranscriptional level, AML cell lines without SOCS1 promoter methylation were used in subsequent studies. Compared to bone marrow cells from healthy donors, four AML cell lines, KG-1, HEL, ML-1 and ML-2, presented lower protein levels and had no SOCS1 promoter methylation. More importantly, HEL cell line had the lowest CUEDC2 protein level; whereas KG-1 cells had the highest CUEDC2 protein level among these four cell lines. Therefore, HEL and KG-1 cells were used to overexpression and knockdown of CUEDC2 expression, respectively. The expression of CUEDC2 increased 5–6-fold in CUEDC2 overexpressed cells, while the expression of CUEDC2 in CUEDC2 knockdown cells only had about 10% of those in WT KG-1 cells (Fig. 3a, b).

Luciferase assays suggested that CUEDC2 overexpression inhibited IL-6-induced STAT3 activation (Fig. 3c). In contrast, higher levels of IL-6-induced STAT3 activation were observed in CUEDC2 knockdown cells than that in control cells (Fig. 3d). Furthermore, an analysis of IL-6-induced STAT3 phosphorylation over time also suggested that CUEDC2 overexpression significantly inhibited STAT3 and JAK1 phosphorylation (Fig. 3e). However, CUEDC2 knockdown enhanced JAK1 and STAT3 phosphorylation in KG-1 cells (Fig. 3f). More interestingly, no obvious decrease of total JAK1 was observed except IL-6 stimulation at 60 min with CUEDC2 overexpressed. The similar result on changes of total JAK1 proteins was also reported previously^19^.

### CUEDC2 inhibited the proliferation of AML cell lines

**Fig. 4:**
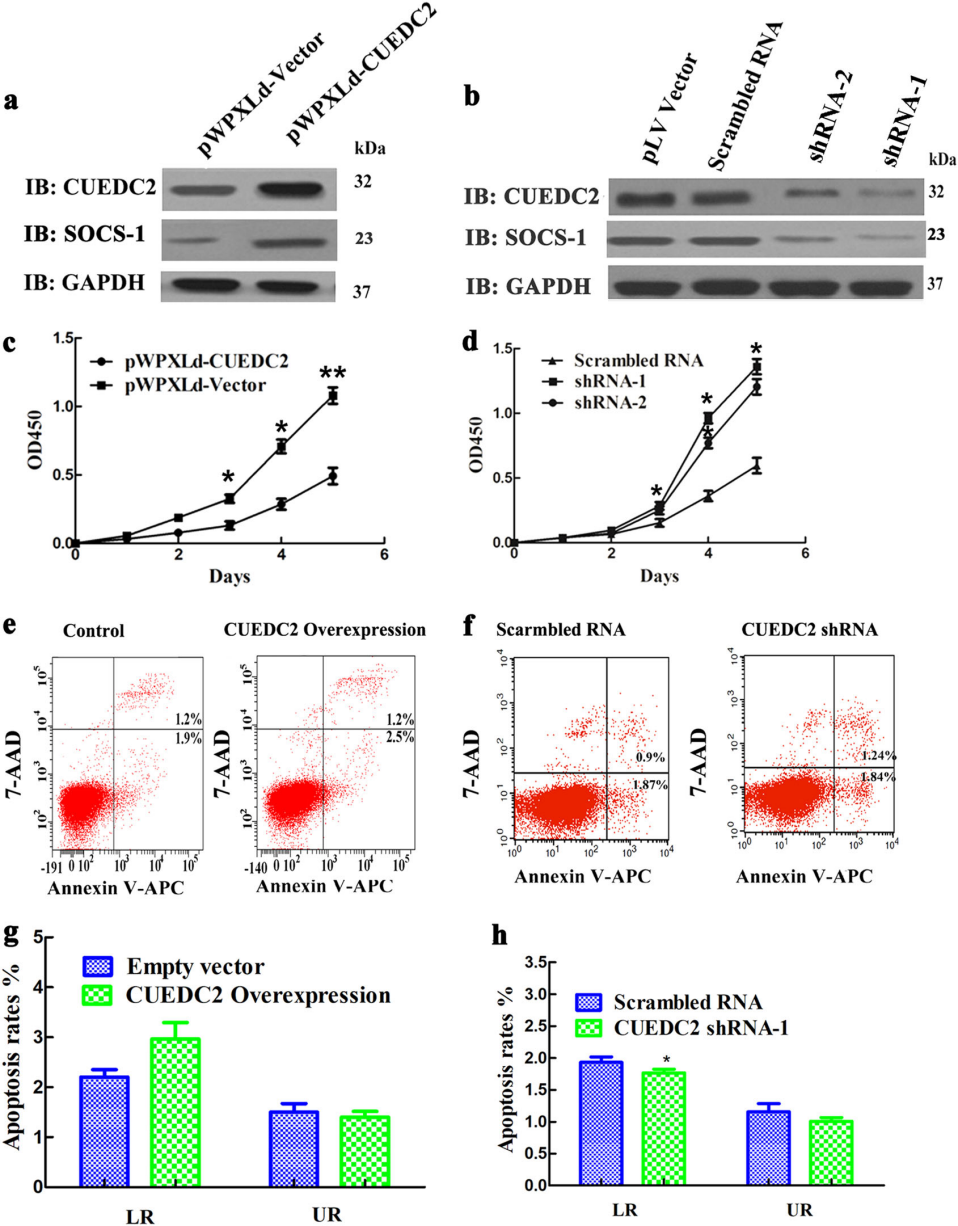
Effects of the overexpression or knockdown of CUEDC2 on the proliferation and apoptosis of HEL and KG-1 cells. **a** and **b** Western blots showing the efficacy of CUEDC2 overexpression and knockdown in HEL and KG-1 cells. SOCS1 expression was also simultaneously determined using western blotting. **c** and **d** A CCK-8 assay was performed to evaluate the effects of CUEDC2 overexpression or knockdown on the proliferation of HEL and KG-1 cells. **e** and **g** Flow cytometry was used to determine effects of CUEDC2 overexpression on the apoptosis of HEL cells. **f** and **h** Flow cytometry was used to determine the effects of CUEDC2 knockdown on the apoptosis of KG-1 cells. All experiments were conducted at least three times. *P *<* 0.05, **P *<* 0.01

HEL and KG-1 cells in which CUEDC2 stably overexpressed or knockdown were obtained by lentivirus-mediated transfection. Western blots revealed an approximately fivefold increase in CUEDC2 expressed HEL cells (Fig. 4a), but CUEDC2 expression in shRNA-1-transfected KG-1 cells was reduced to about 10% of that levels in wild-type (WT) cells (Fig. 4b). More importantly, SOCS1 expression was also increased or decreased with overexpression or knockdown of CUEDC2 in HEL and KG-1 cells, respectively (Fig. 4a, b). Overexpression of CUEDC2 significantly inhibited the proliferation of HEL cells (Fig. 4c), while knockdown the expression of CUEDC2 promoted the proliferation of KG-1 cells (Fig. 4d). Combined with aforementioned results, we deduced that overexpression of CUEDC2 inhibited the proliferation of AML cells, possibly by suppressing the activity of JAK1-STAT3 pathway.

### CUEDC2 had no effects on the proportions of apoptotic AML cells

No significant differences in proportions of late apoptotic and total apoptotic cells were observed between CUEDC2 overexpressed HEL cells and control cells. Whereas knockdown the expression of CUEDC2 in KG-1 cells decreased the proportion of early apoptotic cells compared with that in control cells (Fig. 4e–h). This result was consistent with previous study in the K562 cells^20^.

### CUEDC2 regulated the cell cycle of AML cell lines

**Fig. 5:**
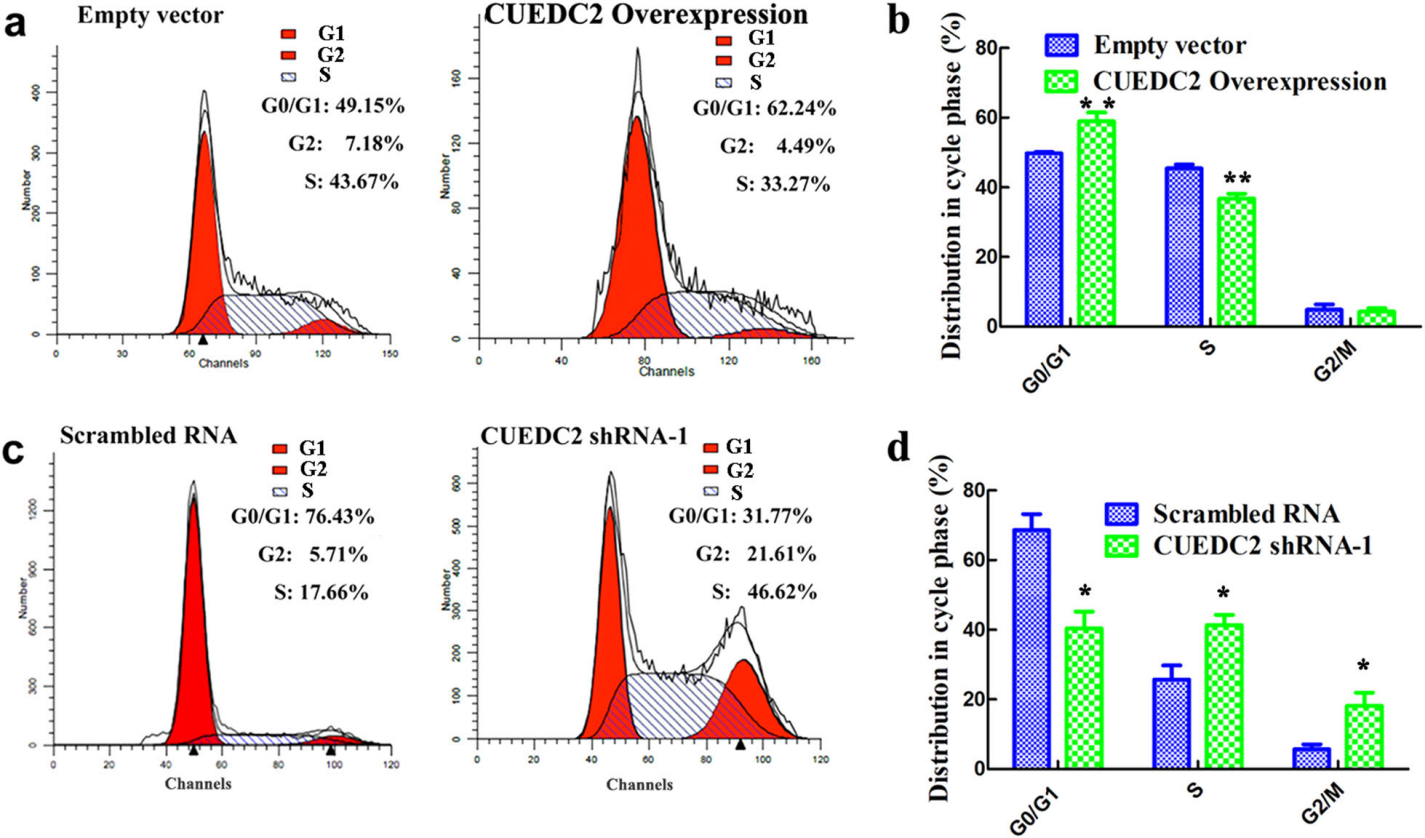
Impact of CUEDC2 on the cell cycle transitions of AML cells. **a** and **b** Effects of CUEDC2 overexpression on the cell cycle progression and distribution of HEL cells in different phases of the cell cycle. **c** and **d** Effects of CUEDC2 knockdown on cell cycle progression and the distribution in KG-1 cells in different phases of the cell cycle. The experiments were conducted at least three times. *P *<* 0.05, **P *<* 0.01

Effects of CUEDC2 on the cell cycle transitions of AML cells were determined. In the presence of overexpressed CUEDC2, a greater percentage of HEL cells were in G0/G1 phase, suggesting that overexpression of CUEDC2 caused G1 phase arrest (Fig. 5a, b). In contrast, knockdown the expression of CUEDC2 in KG-1 cells increased the percentage of cells in S and G2/M phase but reduced the percentage of cells in G0/G1 phase (Fig. 5c, d).

### Effects of CUEDC2 on the sensitivity of AML cell lines to cytarabine and idarubicin

**Fig. 6:**
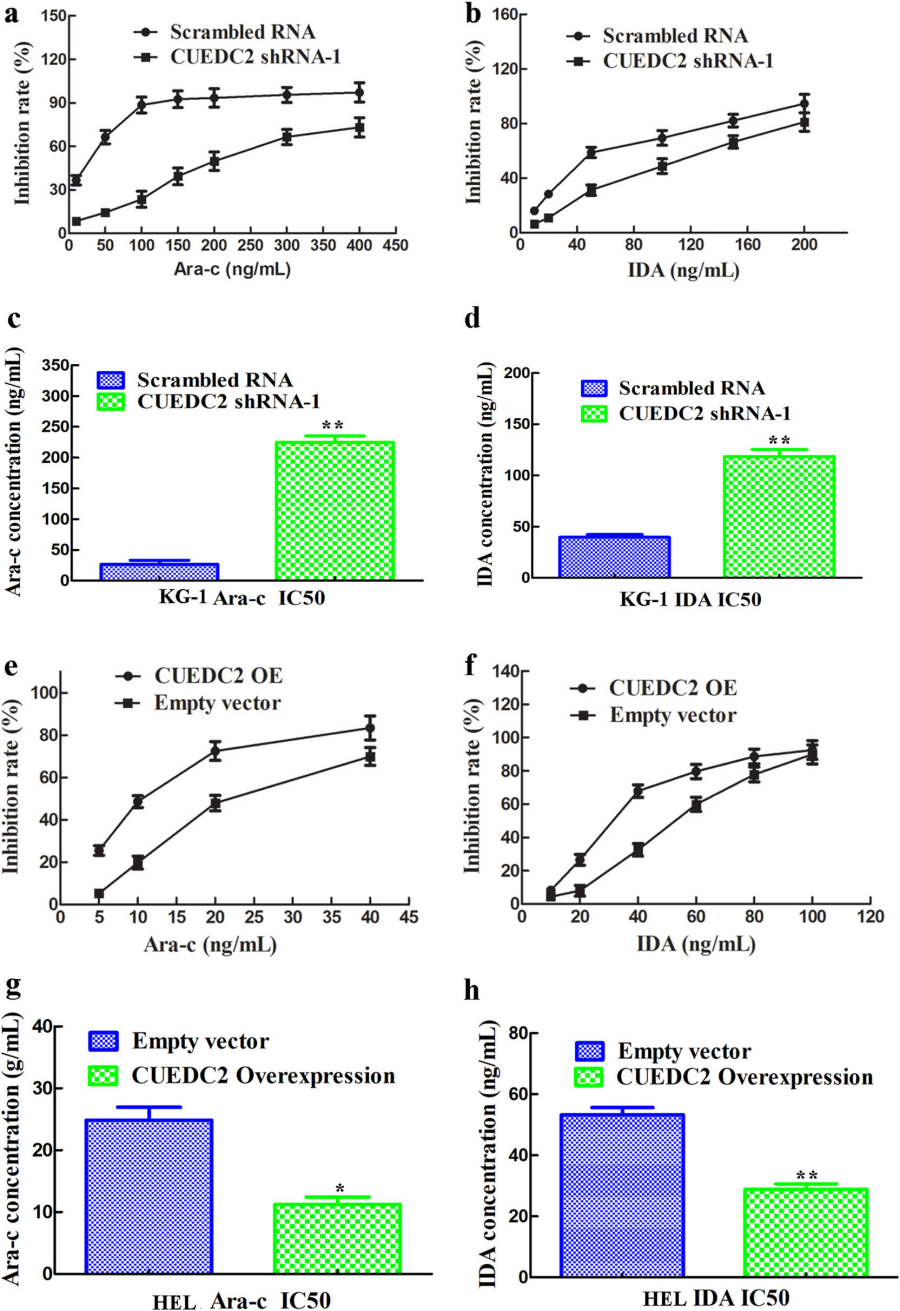
CUEDC2 knockdown in KG-1 cells increased their sensitivity to and the half-maximal inhibitory concentrations of cytarabine and idarubicin, whereas CUEDC2 overexpression in HEL cells decreased their sensitivity to and the half-maximal inhibitory concentrations of cytarabine and idarubicin. **a** and **b**, CCK-8 experiments were performed to analyse effects of CUEDC2 knockdown on the sensitivity of KG-1 cells to cytarabine and idarubicin. **c** and **d** CUEDC2 knockdown in KG-1 cells increased the half-maximal inhibitory concentrations of cytarabine and idarubicin. **e** and **f** CCK-8 experiments were performed to analyse the effects of CUEDC2 overexpression on the sensitivity of HEL cells to cytarabine and idarubicin. **g** and **h** CUEDC2 overexpression in HEL cells decreased the half-maximal inhibitory concentrations of cytarabine and idarubicin. Experiments were conducted at least three times. *P *<* 0.05, **P *<* 0.01

Effects of knockdown or overexpression of CUEDC2 on the sensitivity of AML cells to chemotherapeutic drugs was determined. When CUEDC2 was knockdown in KG-1 cells, its resistance to cytarabine and idarubicin was significantly increased (Fig. 6a, b). At the same time, the half-maximal inhibitory concentrations of them were also increased (Fig. 6c, d). Meanwhile, in the presence of overexpressed CUEDC2 in HEL cells, it became more sensitivity to cytarabine and idarubicin than that of control cells (Fig. 6e, f). Similarities, its half-maximal inhibitory concentrations of them were also decreased (Fig. 6g, h).

### CUEDC2 knockout promoted the progression of AML and shortened latencies in a mouse model of AML

**Fig. 7:**
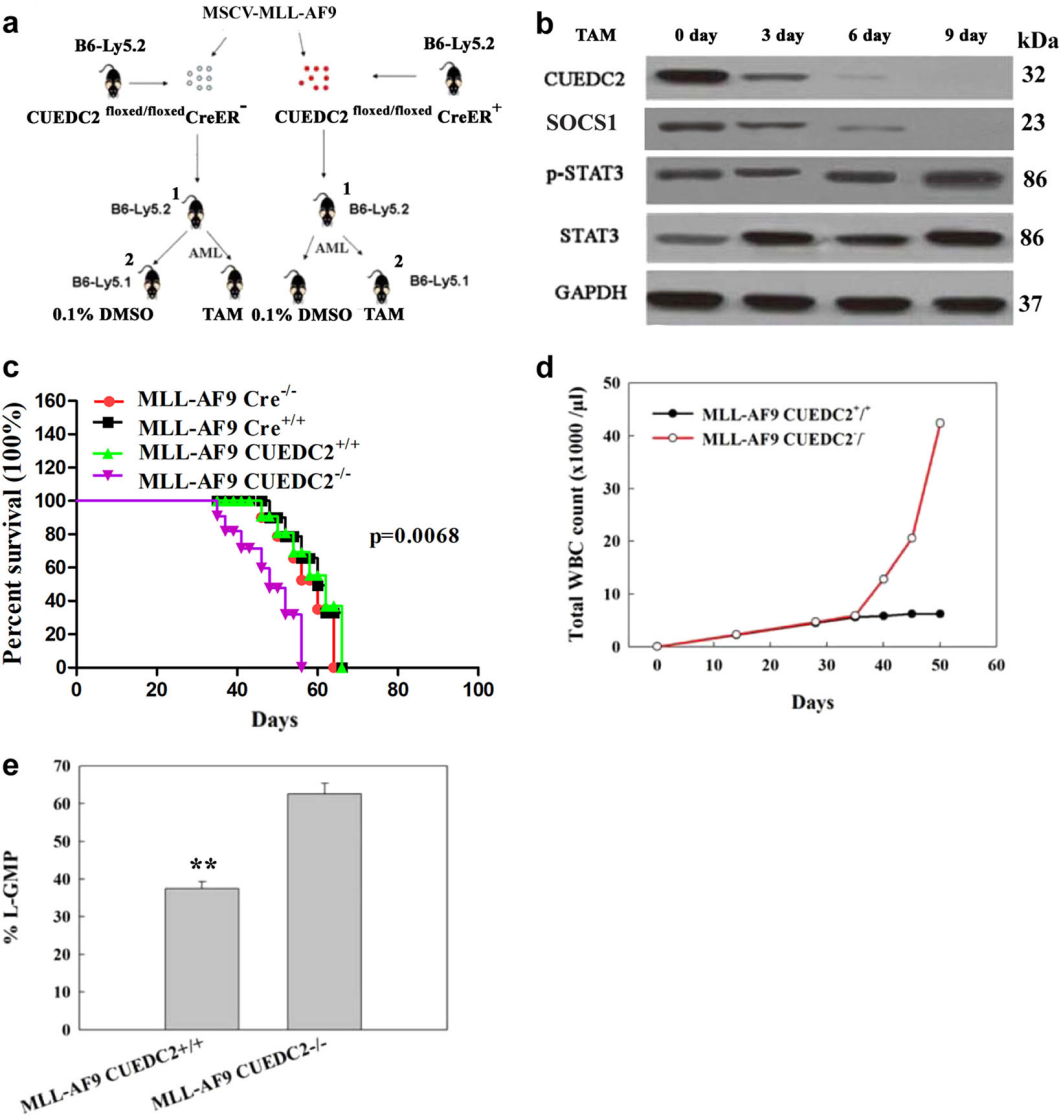
CUEDC2 deficiency promoted MLL-AF9-induced leukaemia in vivo. **a** Schematic of the experiment in which MLL-AF9^+^ CUEDC2^floxed^ murine (BM) cells recovered from a leukaemic primary recipient mouse carrying (Cre^+^) or lacking the CreER transgene (Cre^−^) were transplanted into recipient mice. Cre^+^ and Cre^−^ mice were administered PBS containing 0.1% DMSO or TAM, and then the leukaemia burden was examined in Cre^+^ and Cre^−^ mice (day 30 for Cre^−^ mice and day 36 for Cre^+^ mice). **b** Leukaemic BM cells were isolated from Cre^+^ mice that had been administered TAM for approximately 9 days; the efficiency of CUEDC2 knockout and its effects on the expression of SOCS1 and the activity of the JAK1-STAT3 signalling pathway were determined at 3, 6 and 9 days. **c** and **d** Kaplan-Meier survival curve analysis of mice transplanted and treated as described above (C: *P* = 0.4718; *n* = 12 and D: *P* = 0.0068; n = 15). **e** Analysis of the WBC counts in the peripheral blood collected from Cre^+^ mice treated with PBS containing 0.1% DMSO or TAM-treated mice daily for 4–14 days (*n* = 10). (f) Leukaemic BM cells isolated from Cre^+^ mice administered PBS containing 0.1% DMSO or TAM 7 days earlier were analysed for the mean proportions ± SEM of L-GMPs (*P* = 0.0095; *n* = 10)

MLL-AF9 bone marrow transplant experiments were performed to determine whether CUEDC2 was involved in MLL-AF9-induced AML in vivo. A detailed description of protocols was summarised in Fig. 7a. The efficiency of tamoxifen (TAM)-induced CUEDC2 knockout was detected. On the sixth day after TAM injection, CUEDC2 expression was no longer detected (Fig. 7b). More importantly, the SOCS1 expression was simultaneously decreased as CUEDC2 expression decreased (Fig. 7b). Moreover, increased activation of JAK1-STAT3 pathway was observed over time after the TAM injection (Fig. 7b).

Then effects of CUEDC2 knockdown on the AML mouse latencies and the AML progression were also investigated. Recipient mice transplanted with Cre-expressing leukaemia cells (without CUEDC2^floxed^) or mice transplanted with TAM-treated Cre^+^ cells (without CUEDC2^floxed^) had similar latencies to AML development (Fig. 7c). Mice transplanted with TAM-treated Cre^+^ leukaemia cells (CUEDC2 deleted) displayed a significantly shorter latency (Fig. 7c). Moreover, TAM-treated Cre^+^ transplanted mice showed higher white blood cell (WBC) counts than that of WT mice (Fig. 7d). Because the leukaemia-granulocyte macrophage progenitor (L-GMP) population of this AML model was enriched for LSCs^21^, we also assessed whether the CUEDC2 deficiency affected L-GMP frequency. The CUEDC2 deficiency increased the percentage of L-GMPs in the bone marrow (Fig. 7e).

### Low expression of CUEDC2 indicated low OS and EFS rates for patients with AML

CUEDC2 expression and its correlation with OS and EFS in 188 AML patients were analysed. Our result indicated that low-CUEDC2 expression in AML patients displayed low-OS and -EFS rates, when compared with AML patients with high CUEDC2 expression (Fig. S1).

### CUEDC2 stabilised SOCS1 and protected it from degradation by enhancing SOCS1-Elongin C-CUL2 interaction

**Fig. 8:**
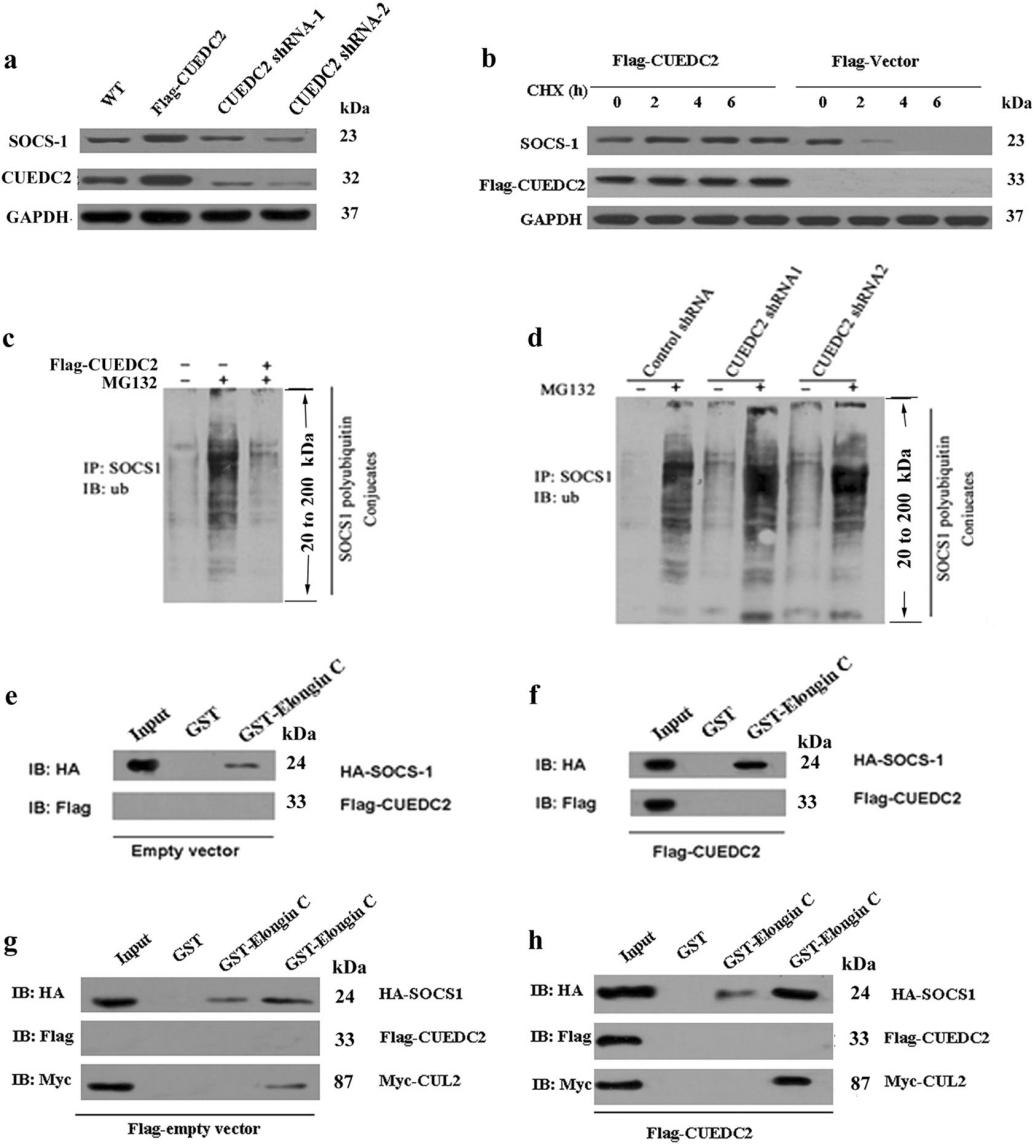
CUEDC2 stabilised SOCS1 by protecting it from degradation through enhanced interactions with Elongin C and CUL2. **a** HEK293T cells were transfected with Flag-CUEDC2 and the CUEDC2 shRNA (shRNA-1 and shRNA-2); untransfected cells were used as control. Cell lysates were subjected to immunoblotting (IB) using the indicated antibodies. **b** Flag-CUEDC2 or Flag vectors were transfected into HEK293T cells. Sixteen hour after transfection, cells were treated with the protein synthesis inhibitor cycloheximide, harvested at the indicated times, and then levels of the SOCS1 protein were analysed by immunoblotting. **c** and **d**HEK293T cells were transfected with Flag-CUEDC2 and the CUEDC2 shRNA; the Flag vector and the scrambled siRNA were used as controls. Cells were then treated with or without the proteasome inhibitor MG132 for an additional 6 h. Cell lysates were immunoprecipitated with an anti-SOCS1 antibody and detected with an ubiquitin antibody. **e** and **f** HEK293T cells were cotransfected with Flag-CUEDC2 (or Flag vector) and HA-SOCS1, cell lysates were prepared, and the inputs were normalised. After an incubation with purified GST or the GST-Elongin C fusion protein and extensive washes, bound proteins were analysed by immunoblotting with anti-Flag and anti-HA antibodies. **g** HEK293T cells were cotransfected with Flag vector, HA-SOCS1 and Myc-CUL2, cell lysates were prepared, and inputs were normalised. After an incubation with purified GST or the GST-Elongin C fusion protein and extensive washes, bound proteins were analysed by immunoblotting with anti-Flag, anti-HA and anti-Myc antibodies. **h** HEK293T cells were cotransfected with Flag-CUEDC2, HA-SOCS1 and Myc-CUL2, cell lysates were prepared, and inputs were normalised. After an incubation with purified GST or the GST-Elongin C fusion protein and extensive washes, bound proteins were analysed by immunoblotting with anti-Flag, anti-HA and anti-Myc antibodies

Based on our results, CUEDC2 was important for the leukaemogenesis of AML by inhibiting JAK1-STAT3 pathway, but how CUEDC2 regulated these processes remains unclear. So, whether CUEDC2 regulated the stability of SOCS1 was detected. As expected, overexpression of CUEDC2 increased SOCS1 protein level, whereas knockdown the expression of CUEDC2 significantly decreased SOCS1 protein level (Fig. 8a). Thus, the SOCS1 protein level depended on CUEDC2 expression. The cycloheximide treatment was used to identify the mechanism might be responsible for CUEDC2-mediated increase in SOCS1 protein level. Significantly higher levels of SOCS1 protein were detected in CUEDC2-overexpressing cells than that in control cells (Fig. 8b). Moreover, levels of SOCS1 protein decreased in control cells over time, and the protein was not detectable after a 6 h treatment (Fig. 8b). Thus, CUEDC2 might increase the expression of SOCS1 by increasing its stability.

Then, how CUEDC2 regulated the ubiquitin-mediated degradation of SOCS1 was analysed. Our results revealed that overexpression of CUEDC2 decreased the amount of ubiquitinated SOCS1 in the presence of MG132 (Fig. 8c), whereas knockdown the expression of CUEDC2 increased levels of ubiquitinated SOCS1 (Fig. 8d). Thus, CUEDC2 stabilised SOCS1 by attenuating its ubiquitination.

Interactions between SOCS family members and Elongin B/C have been shown to limit their turnover^9,13^, any process reduced these interactions promoted the degradation of SOCS proteins. Therefore, we next determined whether CUEDC2 affected the interaction between SOCS1 and Elongin B/C. As shown in Fig. 8e, f, a greater amount of SOCS1 was pulled down in the presence of overexpressed CUEDC2 compared to the input. Thus, although CUEDC2 did not recruit SOCS1 to Elongin C, the interaction between CUEDC2 and SOCS1 might promote the interaction between SOCS1 and Elongin C to attenuate SOCS1 ubiquitination.

According to Zhang et al.^9^, CUL2 might enhance the Elongin B/C and SOCS1 interaction, and subsequently increase the stability of SOCS1. HA-SOCS1 was coexpressed with Myc-CUL2 (Myc-vector) and Flag-CUEDC2 (Flag vector) in HEK293T cells, and a greater amount of SOCS1 was pulled down in the presence of overexpressed CUEDC2 and CUL2 (Fig. 8h) than that in the input, cells expressing SOCS1 or CUL2 alone (Fig. 8g) or cells coexpressing SOCS1 and CUEDC2 (Fig. 8f). Thus, our data suggested that CUEDC2 enhanced the SOCS1-Elongin C-CUL2 interaction, reduced SOCS1 ubiquitination, and facilitated SOCS1 stabilisation.

## Discussion

The promoter hypermethylation and elevated ubiquitin-mediated degradation caused down-regulation of SOCS1 was reported as main reasons for JAK1-STAT3 activation and leukaemogenesis of AML^6,7^. But detailed regulating mechanism of SOCS1 degradation is still not fully known. Thus, exploration the regulating mechanism of ubiquitin-mediated degradation of SOCS1 in AML is of great important. In this study, a novel ubiquitin-mediated degradation regulator of SOCS1, CUEDC2, and its roles in the leukaemogenesis of AML was reported. Furthermore, we discovered that CUEDC2 exerted its anti-AML roles via attenuated ubiquitin- mediated degradation of SOCS1 by enhancing interactions among SOCS1, Elongin C and CUL2. The enhanced SOCS1 expression inhibited the activation of JAK1-STAT3 pathway and leukaemogenesis of AML.

Although it is generally accepted that SOCS1 is degraded by the proteasome-mediated pathway^6,7^, which protein was involved in regulating the ubiquitin-mediated degradation of SOCS1 remains unclear. In the current study, CUEDC2, a novel interacting partner and a potential regulator of the ubiquitin-mediated degradation of SOCS1, was identified by MS. Further, biochemical studies revealed a direct interaction between the CUE domain of CUEDC2 and SH2 domain of SOCS1. Identified as ubiquitin-binding motifs, CUE domains interact with both mono and polyubiquitin and play dual roles in recognising mono and polyubiquitin as well as in facilitating intramolecular monoubiquitination^14,17^. Thus, CUEDC2 might be a novel regulator of SOCS1’s ubiquitin-mediated degradation. More importantly, correlation study indicated that low CUEDC2 expression exhibited a direct positive correlation with the ubiquitin-mediated degradation of SOCS1 in AML cells without SOCS1 promoter methylation.

Interestingly, a novel mechanism CUEDC2 regulating SOCS1 ubiquitin-mediated degradation is also investigated. The SOCS box-mediated interactions between SOCS proteins and Elongin C have been reported played key roles in regulating proteasome-mediated SOCS proteins degradation^22,23^. Our study showed that CUEDC2 overexpression enhanced SOCS1 and Elongin C interaction, reduced ubiquitin-mediated degradation of SOCS1. However, this result is not completely consistent with previous study; Zhang et al.^19^ showed that CUEDC2 only reduced SOCS3 ubiquitination by promoting SOCS3 and Elongin C interaction. In this study, CUEDC2 regulated both the stability of SOCS1 and SOCS3. Interestingly, no direct interaction between CUEDC2 and Elongin C was observed. Thus, the interaction between CUEDC2 and SOCS1 might promote the interactions among SOCS1, Elongin C and CUL2 by altering the conformational of SOCS1.

Many studies suggested that SOCS1 inhibited cytokine signalling by binding to JAKs and suppressing their catalytic activity^24^ or by SOCS box-mediated proteasomal degradation of JAKs^8,9,23^. Here, we demonstrated that CUEDC2 interacted with SOCS1 and acted as a novel SOCS1 co-operator to inhibit JAK1-STAT3 pathway activation. This might provide a novel potential mechanism of SOCS1-mediated suppression of the JAK1-STAT3 pathway.

Previous studies showed that CUEDC2 deregulation may contribute to tumour initiation by causing chromosomal instability^16,25^. However, the inhibitory role of CUEDC2 in NF-κB activation seems contradictory to its cancer-promoting function as a cell cycle regulator during tumorigenesis^15,26^. Our data supported CUEDC2 as a tumour suppressor because we found that overexpression of CUEDC2 inhibited AML cell proliferation, caused G1 arrest and prolonged the latencies of AML mouse in vitro and in vivo. Besides, clinical studies also demonstrated that low CUEDC2 expression indicating low-OS and -EFS rates in AML patients. Although roles of CUEDC2 in the tumorigenesis of many cancers were studied^14–16,25,27^, controversy remains regarding whether CUEDC2 functions as a tumour suppressor gene or oncogene.

Collectively, CUEDC2 was identified as a novel regulator of ubiquitin-mediated degradation of SOCS1 and an inhibitor of JAK1-STAT3 pathway that inhibited this pathway by reducing the ubiquitin-mediated degradation of SOCS1 via promoting SOCS1, Elongin C and CUL2 interactions. Interactions between CUEDC2 and SOCS1 might promote interactions among SOCS1, Elongin C and CUL2 by altering the conformational of SOCS1. The low CUEDC2 expression in AML led to SOCS1 degradation, increased JAK1-STAT3 pathway activation, and promoted leukaemogenesis of AML. Thus, this study not only found a novel regulator of ubiquitin-mediated degradation of SOCS1 but also elucidated roles and mechanisms of CUEDC2 in leukaemogenesis of AML.

## Materials and methods

### Plasmids, cell culture, mononuclear cells isolated from AML patients and mouse generation

The AML cell lines HEL, U937, THP-1, KG-1, K562, HL-60, ML-1 and ML-2 used in the present study are preserved in our laboratory. For primary AML cells, leukaemic blasts were obtained from the peripheral blood of AML patients after obtaining informed consent. These studies have been sanctioned by the Investigational Review Board of Xuzhou Medical University, and all patients provided informed consent. Mononuclear cells were isolated as previously described^28^. In each case, the percentage of blasts in the peripheral blood was >70%. Blood was collected into heparinized syringes, diluted 1:3 with Roswell Park Memorial Institute (RPMI) 1640 medium, and transferred as an overlayer to centrifuge tubes containing 10 ml of Ficoll-Hypaque (specific gravity, 1.077–1.081, Sigma, St Louis, USA). After centrifugation at room temperature for 30 min, the interface layer, containing predominantly leukaemic blasts, was extracted with a sterile Pasteur pipette, suspended in RPMI medium, and washed three times. These mononuclear cells isolated from patients with AML were used to detect SOCS1 and CUEDC2 expression. Luciferase activities were determined using a Dual-Luciferase Reporter Assay System (Promega). The MSCV-MLL-AF9 plasmid was a kind gift from Dr. Tao Cheng (Chinese Academy of Medical Sciences & Peking Union Medical College). The CUEDC2^floxed^, CreER^+^ mice were generated by Guangzhou Cyagen Biosciences Inc.

### Real-time PCR and methylation-specific polymerase chain reaction

Total RNA was isolated from AML cell lines and mononuclear cells isolated from patients with AML using Trizol reagent (Invitrogen). Reverse transcription was performed with the Transcriptor First Strand cDNA Synthesis Kit (Roche). Primers and TaqMan probes for SOCS1 were purchased from Life Technologies (Gaithersburg, MD). Levels of specific mRNAs in AML cells were detected. The methylation of the SOCS1 promoter in AML cell lines and primary AML cells was detected using a methylation-specific polymerase chain reaction, as previously described^16^.

### Detection of the levels of the SOCS1 and CUEDC2 proteins in AML cells

Levels of the SOCS1 and CUEDC2 proteins in the AML cell lines were detected by western blotting. However, levels of the SOCS1 and CUEDC2 proteins in the mononuclear cells isolated from patients with AML were detected using both the protein expression antibody array protein chip from Shanghai H. Wayen Biotechnology Inc., and western blotting. Levels of the SOCS1 and CUEDC2 protein in the mononuclear cells isolated from the healthy donors were set to 1.0. The decrease in levels of CUEDC2 and SOCS1 proteins in AML cells was used to determine the correlation between the two by calculating Pearson’s correlation coefficient.

### Construction and production of lentiviruses

Lentiviral pLV-shRNA and pWPXLd plasmids were used to knockdown and overexpress CUEDC2 in AML cells. Two CUEDC2-targeting sequences were designed by BLOCK-iT^™^ RNAi Designer from Life Technologies. Sequences of the CUEDC2 shRNA, scrambled RNA and pWPXLd-CUEDC2 are listed in Table S3. The viruses were propagated in HEK293T cells by cotransfecting cells with the corresponding plasmids along with the helper plasmids pSPXA2 and pMD2.G. After 72 h incubation, the supernatant was collected and concentrated by ultracentrifugation.

### Establishment of stable cell lines

AML cell lines were transfected with the CUEDC2 shRNA, pWPXLd-CUEDC2 or control lentiviruses for 72 h. Next, cells were continuously cultured in the medium containing 1.2 μg/mL puromycin for approximately 15 days. Finally, AML cells transfected with CUEDC2, shRNA or control lentiviruses were sorted by flow cytometry.

### Immunoprecipitation, immunoblotting and mass spectrometry

Total proteins were extracted using Mammalian Protein Extraction Reagent (Thermo) supplemented with a complete protease inhibitor cocktail (Roche). Immunoprecipitations were performed by incubating whole cell extracts with the indicated antibody for 6 h at 4 °C with rocking after a preincubation with Protein A/G-Sepharose (Abcam). Immunoprecipitates were washed and resolved by SDS-PAGE. For MS, after Coomassie Brilliant blue staining (Sigma-Aldrich), total protein bands in the lane were excised and analysed by ion-trap MS at Shanghai Genechem Co., Ltd. Antibodies against Flag (No. 8146), Myc (No. 2272), anti-HA (No. 3724), SOCS1 (No. 3950), STAT3 (No. 9139), pSTAT3 (No. 9131), JAK1 (No. 3332), pJAK1 (No. 3331) and Ub (No. 3936) were purchased from Cell Signalling Technology.

### Cell viability assay

Cell proliferation was assessed with the Cell Counting Kit-8 (CCK-8) assay. AML cells were seeded into 96-well plates at a density of 3 × 10^3^ cells per well, and viability was detected every 24 h for 5 days. Approximately, 2.0 × 10^4^ cells were treated with various concentrations of cytarabine and idarubicin for 48 h, and 10 μL of CCK-8 were then added to each well to determine the cells’ sensitivity to chemotherapeutic drugs. Following 4 h incubation, the absorbance was measured at 450 nm.

### Analysis of the cell cycle and apoptosis by flow cytometry

Cells were plated in 6-well plates at a density of 2 × 10^6^ cells and cultured in 2 mL of serum-free IMDM medium for 24 h to synchronise the cell cycle for cell cycle analysis. The phases of the cell cycle were determined by staining cells with 7-AAD (Sigma), according to the manufacturer’s protocols. AML cell apoptosis was detected using an Annexin-V-APC and 7-AAD kit, according to the manufacturer’s instructions. The analysis was performed on a FACScan cytometer (BD) using CellQuest software.

### GST pulldown assay

GST and GST fusion proteins were expressed in the *Escherichia coli* BL21 strain and purified according to manufacturer’s instructions (GE Healthcare). The Myc-SOCS1 protein, which was obtained from the whole cell lysates of the indicated cells transfected with the Myc-SOCS1 plasmid, was incubated with GST and GST-CUEDC2 or its truncated fusion protein bound to GST beads in 1 mL of binding buffer containing protease inhibitor cocktail at 4 °C for 6 h. GST beads were then washed three times, suspended in 20 μL of 1× SDS-PAGE loading buffer and detected by immunoblotting.

### Effects of CUEDC2 knockout on the survival of the AML mouse model

Both CUEDC2^floxed/floxed^ CreER^−^ and CUEDC2^floxed/floxed^ CreER^+^ mice were administered 150 mg/kg 5-fluorouracil (5-FU, Sigma). Recovered mononuclear bone marrow cells (MNBCs) were transduced with recombinant MLL-AF9-expressing retroviruses and transplanted into lethally irradiated F1 C57BL6, Ly-45.2 mice. Recipient mice developed leukaemia within 40–60 days, with a median survival time of 51 days. The survival rates of the different groups were determined.

### Statistical analysis

Statistical analyses were performed using the SPSS version 16.0. Data are presented as the means ± SEM of three independent experiments. The log-rank test was used to determine *P* values for all Kaplan–Meier survival curve analyses. The correlations between the levels of the CUEDC2 and SOCS1 proteins in AML cells were analysed by calculating Pearson’s correlation coefficients. Comparisons of mean values between the control and treated groups were analysed using Student’s *t* test. *P*  < 0.05 was considered significant.

## Electronic supplementary material

Figure S1

Table S1

Table S2

Table S3

Summary and Figure legend for supplemnbtary date
