# Supplementary figures and images for: CUEDC2, a novel interacting partner of the SOCS1 protein, plays important roles in the leukaemogenesis of acute myeloid leukaemia
Source: Cell Death Dis. 2018 Jul 10;9(7):774. doi: 10.1038/s41419-018-0812-6 (PMC6039501; doi:10.1038/s41419-018-0812-6)

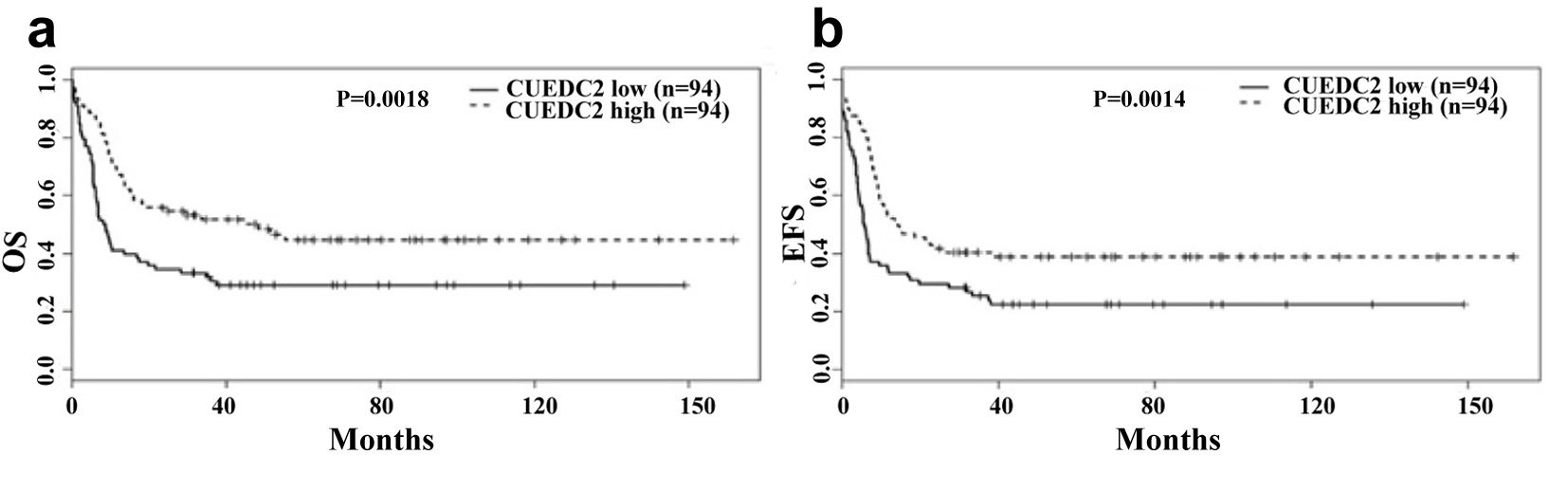

Supplement: Supplementary file 1 — Figure S1 [file 41419_2018_812_MOESM1_ESM.jpg]
