# Supplementary material for: CUEDC2, a novel interacting partner of the SOCS1 protein, plays important roles in the leukaemogenesis of acute myeloid leukaemia
Source: Cell Death Dis. 2018 Jul 10;9(7):774. doi: 10.1038/s41419-018-0812-6 (PMC6039501; doi:10.1038/s41419-018-0812-6)
Supplement: Supplementary file 2 — Table S1 [file 41419_2018_812_MOESM2_ESM.doc]

**Table S1 SOCS1-interacting proteins identified by MS/MS.**

| **Accession number** | **Gene name** | **Protein name** | **Uni. Pep** | **Coverage（%）** |
| --- | --- | --- | --- | --- |
| IPI00011218 | CSF1R | Macrophage colony-stimulating factor 1 receptor | 87 | 78.1 |
| IPI00015881 | CSF1 | Macrophage colony-stimulating factor 1 | 54 | 54.3 |
| IPI00018274 | EGF receptor | Epidermal growth factor receptor | 64 | 62.1 |
| IPI00005722 | FLT3 | Receptor-type tyrosine-protein kinase FLT3 | 39 | 43.8 |
| **IPI00027969      IPI00910957** | **VHL** | **Von Hippel-Lindau Tumor Suppressor** | **18** | **45.8** |
| **IPI00300341   IPI00791185** | **ELOC** | **Elongin-C** | **32** | **88.5** |
| **IPI00410162;IPI00026670;IPI00917694** | **ELOB** | **Elongin-B** | **20** | **65.7** |
| IPI00216003 | CUL5 | Cullin 5 | 6 | 12.6 |
| **IPI00014311   IPI00985090** | **CUL2** | **Cullin 2** | **26** | **77.4** |
| IPI00003386 | RBX1 | E3 ubiquitin-protein ligase RBX1 | 12 | 22.6 |
| [IPI00107502](http://www.dkfz.de/menu/cgi-bin/srs7.1.3.1/wgetz?-newId+%5BIPI-AllText:IPI00107502%5D%26%5BIPI-NCBI_TaxID:9606%5D+-lv+30+-view+SeqSimpleView+-page+qResult) | WSB1 | WD repeat and SOCS box-containing protein 1 | 17 | 25.6 |
| **IPI00100463** | **CUEDC2** | **CUE domain-containing protein 2** | **46** | **81.3** |
| IPI01019055 | LRRC41 | Leucine Rich Repeat Containing 41 | 12 | 33.6 |
| IPI00386448 | RELA | Transcription factor p65 | 11 | 18.9 |
| IPI00171117 | COMMD1 | COMM domain-containing protein 1 | 16 | 34.6 |
| IPI00011736 | PIK3R2 | Phosphoinositide-3-Kinase Regulatory Subunit | 12 | 18.9 |
| IPI00464978 | IRS2 | insulin receptor substrate 2 | 33 | 22.7 |
| IPI00296992 | AXL | Tyrosine-protein kinase receptor UFO | 15 | 45.8 |
| IPI00000044 | PDGFB | Platelet-derived growth factor subunit B | 12 | 26.6 |
| IPI00289207 | TRIM8 | E3 ubiquitin-protein ligase TRIM8 | 8 | 10.2 |
| IPI00472451 | VAV2 | Guanine nucleotide exchange factor VAV2 | 34 | 21.8 |
| IPI00011696 | VAV1 | Proto-oncogene vav | 42 | 31.6 |
| IPI00718966 | TIRAP | TIR domain containing adaptor protein | 12 | 21.7 |
| IPI00412829 | TEK | TEK tyrosine kinase | 55 | 33.8 |
| IPI00000878 | TEC | Tyrosine-protein kinase Tec | 32 | 32.3 |
| IPI00332692 | Pim2 | Serine/threonine-protein kinase pim-2 | 21 | 45.4 |
| IPI00021448 | PIK3R1 | Phosphatidylinositol 3-kinase regulatory subunit alpha | 15 | 24.7 |
| IPI00028065 | NCK1 | Cytoplasmic protein NCK1 | 21 | 31.1 |
| IPI00022296 | KIT | Mast/stem cell growth factor receptor | 53 | 42.6 |
| IPI00219417 | JAK3 | Tyrosine-protein kinase JAK3 | 67 | 48.8 |
| IPI00031016 | JAK2 | Tyrosine-protein kinase JAK2 | 58 | 42.4 |
| IPI00784013 | JAK1 | Tyrosine-protein kinase JAK1 | 86 | 63.5 |
| IPI00004566 | ITK | Tyrosine-protein kinase ITK/TSK | 44 | 46.9 |
| IPI00019471 | IRS1 | insulin receptor substrate 1 | 34 | 34.5 |
| IPI00878728 | IL2RB | interleukin 2 receptor subunit beta | 68 | 43.7 |
| IPI00010808 | IFNGR1 | Interferon gamma receptor 1 | 13 | 35.3 |
| IPI00027232 | IGF1R | [Insulin-like growth factor 1 receptor](http://hprd.org/interactions?hprd_id=00975&isoform_id=00975_1&isoform_name=Isoform_1) | 45 | 31.7 |
| IPI00433029 | IGF1 | Insulin-like growth factor I | 51 | 45.9 |
| IPI00295079 | GHRHR | Growth hormone-releasing hormone receptor | 21 | 37.2 |
| IPI00186990 | Grb2 | Growth factor receptor-bound protein 2 | 22 | 58.4 |
| IPI00219012 | Fyn | Tyrosine-protein kinase Fyn | 34 | 61.3 |
| IPI00922415 | SSBP1 | Single-stranded DNA-binding protein | 15 | 21.2 |
| IPI00017726 | HADH2 | Isoform 2 of 3-hydroxyacyl-CoA dehydrogenase type-2 | 14 | 16.5 |
| IPI00645948 | HMGB1 | High-mobility group box 1 | 21 | 34.2 |
| IPI00028159 | CXCR4 | C-X-C chemokine receptor type 4 | 33 | 54.4 |
| IPI00025803 | INSR | nsulin receptor | 22 | 43.4 |
| IPI00027637 | MPL | Thrombopoietin receptor | 44 | 55.7 |
| IPI00216969 | ABL1 | Tyrosine-protein kinase ABL1 | 32 | 34.4 |
| IPI00298306 | [ATM](https://www.ncbi.nlm.nih.gov/gene/472) | Serine-protein kinase ATM | 13 | 32.1 |
| PI00412298 | ATR | Serine/threonine-protein kinase ATR | 14 | 23.6 |
| IPI00411381 | CLEC7A | C-type lectin domain family 7 member A | 12 | 54.3 |
| PI00004839 | CRKL | Crk-like protein | 34 | 65.7 |
| PI00465234 | CSF2RB | Cytokine receptor common subunit beta | 23 | 33.6 |
| IPI00003591 | CYTIP | cytohesin 1 interacting protein | 20 | 34.5 |
| IPI00514638 | DAB1 | reelin adaptor protein | 21 | 43.4 |
| IPI00941732 | ELOA | elongin A | 18 | 65.3 |
| IPI00013159 | ETV6 | ets variant 6 | 21 | 33.5 |
| IPI00027174 | FGFR3 | Fibroblast growth factor receptor 3 | 32 | 22.5 |
| IPI00291901 | IRF3 | interferon regulatory factor 3 | 27 | 27.7 |
| IPI00412433 | MAP3K5 | mitogen-activated protein kinase kinase kinase 5 | 32 | 43.5 |
| IPI00014830 | PDCD1 | programmed cell death 1 | 21 | 31.7 |
| IPI00012587 | PTEN | Phosphatase and Tensin homolog deleted on chromosome Ten | 23 | 35.1 |
| IPI00658023 | PTPN11 | Tyrosine-protein phosphatase non-receptor type 11 | 45 | 22.4 |
| PI00026262 | RASA1 | Ras GTPase-activating protein 1 | 43 | 19.8 |
| IPI00033944 | SOCS2 | Suppressor of cytokine signaling 2 | 21 | 56.3 |
| IPI00025087 | TP53 | Cellular tumor antigen p53 | 36 | 42.3 |
| IPI00743663 | TRAF6 | (TNF receptor associated factor 6 | 55 | 52.7 |
| IPI00873422 | TRIM5 | Tripartite motif-containing protein 5 | 22 | 34.6 |
| IPI00295081 | TUBG1 | Tubulin gamma-1 chain | 21 | 44.8 |
| IPI00022353 | TYK2 | tyrosine kinase 2 | 56 | 42.7 |
| IPI00329132 | ATAT1 | alpha tubulin acetyltransferase 1 | 13 | 9.8 |
|  | Nef | S100 calcium binding protein B | 21 | 11.3 |
| IPI00218019 | BSG | Isoform 1 of Basigin | 14 | 11.2 |
| IPI00301263 | CAND1 | Isoform 1 of Cullin-associated NEDD8-dissociated protein 1 | 36 | 22.1 |
| IPI00219078 | ATP2A2 | Isoform SERCA2B of Sarcoplasmic/endoplasmic reticulum calcium ATPase 2 | 22 | 11.2 |
| IPI00784090 | PTS | 6-pyruvoyl tetrahydrobiopterin synthase | 6 | 6.4 |
| IPI00300341 | TCEB1 | Transcription elongation factor B polypeptide 1 | 21 | 78.3 |
| IPI00099996 | MRPP1 | Mitochondrial ribonuclease P protein 1 | 8 | 6.8 |
| IPI00296337 | CPS1 | carbamoyl-phosphate synthetase 1 isoform a precursor | 4 | 3.3 |
| IPI00019385 | SSR4 | Translocon-associated protein subunit delta precursor | 4 | 4.6 |
| IPI00003519 | EFTUD2 | 116 kDa U5 small nuclear ribonucleoprotein component | 6 | 3.1 |
| IPI00031370 | ARL1 | ADP-ribosylation factor-like protein 1 | 5 | 2.4 |
| IPI00302850 | SNRPD1 | Small nuclear ribonucleoprotein Sm D1 | 3 | 2.1 |
| IPI00216691 | PFN1 | Profilin-1 | 6 | 4.3 |
| IPI00007215 | ASB3 | Isoform 1 of Ankyrin repeat and SOCS box protein 3 | 8 | 12.6 |
| IPI00104050 | THRAP3 | Thyroid hormone receptor-associated protein 3 | 10 | 14.3 |
| IPI00007188 | SLC25A5 | ADP/ATP translocase 2 | 13 | 5.7 |

Potential SOCS1-interacting proteins are listed with their IPI accession numbers. The indicated coverage (in percentage) and the number of unique peptides identified were obtained by LC-MS/MS. The proteins mentioned related to the SOCS1 ubiquitination degradation in this manuscript highlighted by bold.

Abbreviations: Uni. Pep. Unique peptides (number of unique peptides identified per protein); Cov. Coverage.
