# Supplementary material for: CUEDC2, a novel interacting partner of the SOCS1 protein, plays important roles in the leukaemogenesis of acute myeloid leukaemia
Source: Cell Death Dis. 2018 Jul 10;9(7):774. doi: 10.1038/s41419-018-0812-6 (PMC6039501; doi:10.1038/s41419-018-0812-6)
Supplement: Supplementary file 3 — Table S2 [file 41419_2018_812_MOESM3_ESM.doc]

**Table S2 The decreased protein levels of CUEDC2 and SOCS1 in the AML cell lines and different AML subtype primary cells with SOCS1 downregulation caused by ubiquitin degradation and the correlation ratios of the protein levels decreased between them were analyzed by the Pearson correlation method.** (Since the CUEDC2 regulates the downregulation of SOCS1 in the posttranscriptional level, so only AML cell lines and AML primary cells with SOCS1 downregulation by ubiquitin degradation was analyzed.)

**Table S2-1 The decreased protein levels of CUEDC2 and SOCS1 in the AML cell lines with SOCS1 downregulation caused by ubiquitin degradation and the correlation ratio of the protein levels decreased between them were analyzed by the Pearson correlation method.**

| **AML cell line Names with SOCS1 down regulation by ubiquitin degradation** | **The decreased protein levels of CUEDC2 in AML cell lines** | **The decreased protein levels of SOCS1 in AML cell lines** |
| --- | --- | --- |
| KG-1 | 0.56 | 0.62 |
| HEL | 0.86 | 0.88 |
| ML-1 | 0.72 | 0.81 |
| ML2 | 0.62 | 0.61 |
| **Correlation ratio** | **0.946391397** |  |

**Table S2-2 The decreased protein levels of CUEDC2 and SOCS1 in the different AML subtype primary cells with SOCS1 downregulation caused by ubiquitin degradation and the correlation ratios of the protein levels decreased between them were analyzed by the Pearson correlation method.**

| **The AML subtypes** | **The decreased protein levels of CUEDC2 in AML primary cells** | **The decreased protein levels of SOCS1 in AML primary cells** |
| --- | --- | --- |
| **The normal karyotype leukemia patients with SOCS1 downregulation caused by ubiquitin degradation** | **The decreased protein levels of CUEDC2 in normal karyotype AML primary cells** | **The decreased protein levels of SOCS1 in normal karyotype AML primary cells** |
| 1 | 0.42 | 0.48 |
| 2 | 0.24 | 0.28 |
| 3 | 0.18 | 0.29 |
| 4 | 0.36 | 0.37 |
| 5 | 0.44 | 0.59 |
| 6 | 0.56 | 0.71 |
| 7 | 0.72 | 0.89 |
| 8 | 0.35 | 0.33 |
| 9 | 0.52 | 0.48 |
| 10 | 0.48 | 0.61 |
| 11 | 0.29 | 0.26 |
| 12 | 0.28 | 0.25 |
| 13 | 0.35 | 0.49 |
| 14 | 0.62 | 0.61 |
| 15 | 0.33 | 0.28 |
| **Correlation ratio** | **0.914780361** |  |
| **The t(15;17) AML patients with SOCS1 downregulation caused by ubiquitin degradation** | **The decreased protein levels of CUEDC2 in t(15;17) AML primary cells** | **The decreased protein levels of SOCS1 in t(15;17) AML primary cells** |
| 1 | 0.33 | 0.28 |
| 2 | 0.48 | 0.45 |
| 3 | 0.22 | 0.28 |
| 4 | 0.36 | 0.43 |
| 5 | 0.48 | 0.65 |
| 6 | 0.62 | 0.71 |
| 7 | 0.78 | 0.81 |
| 8 | 0.38 | 0.35 |
| 9 | 0.43 | 0.49 |
| 10 | 0.45 | 0.42 |
| 11 | 0.31 | 0.39 |
| 12 | 0.27 | 0.22 |
| **Correlation ratio** | **0.92770792** |  |
| **The complex AML patients with SOCS1 downregulation caused by ubiquitin degradation** | **The decreased protein levels of CUEDC2 in complex AML primary cells** | **The decreased protein levels of SOCS1 in complex AML primary cells** |
| 1 | 0.66 | 0.77 |
| 2 | 0.32 | 0.35 |
| 3 | 0.24 | 0.29 |
| 4 | 0.49 | 0.43 |
| 5 | 0.54 | 0.65 |
| 6 | 0.28 | 0.33 |
| **Correlation ratio** | **0.951946231** |  |
| **The t(11q23)/MLL AML patients with SOCS1 down- regulation caused by ubiquitin degradation** | **The decreased protein levels of CUEDC2 in t(11q23)/MLL AML primary cells** | **The decreased protein levels of SOCS1 in t(11q23)/MLL AML primary cells** |
| 1 | 0.46 | 0.57 |
| 2 | 0.27 | 0.36 |
| 3 | 0.33 | 0.41 |
| 4 | 0.43 | 0.39 |
| 5 | 0.31 | 0.31 |
| 6 | 0.38 | 0.44 |
| 7 | 0.26 | 0.29 |
| 8 | 0.35 | 0.33 |
| 9 | 0.64 | 0.59 |
| 10 | 0.55 | 0.63 |
| 11 | 0.36 | 0.44 |
| 12 | 0.24 | 0.29 |
| 13 | 0.29 | 0.31 |
| 14 | 0.46 | 0.51 |
| 15 | 0.58 | 0.65 |
| 16 | 0.72 | 0.84 |
| 17 | 0.19 | 0.25 |
| 18 | 0.26 | 0.29 |
| 19 | 0.39 | 0.48 |
| 20 | 0.41 | 0.36 |
| 21 | 0.56 | 0.68 |
| **Correlation ratio** | **0.943769992** |  |
| **The t(8;21) AML patients with SOCS1 downregulation caused by ubiquitin degradation** | **The decreased protein levels of CUEDC2 in t(8;21) AML primary cells** | **The decreased protein levels of SOCS1 in t(8;21) AML primary cells** |
| 1 | 0.22 | 0.29 |
| 2 | 0.37 | 0.33 |
| 3 | 0.46 | 0.41 |
| 4 | 0.35 | 0.34 |
| 5 | 0.32 | 0.39 |
| 6 | 0.36 | 0.42 |
| 7 | 0.48 | 0.55 |
| 8 | 0.53 | 0.49 |
| 9 | 0.26 | 0.31 |
| 10 | 0.33 | 0.39 |
| 11 | 0.38 | 0.46 |
| 12 | 0.51 | 0.61 |
| 13 | 0.66 | 0.78 |
| 14 | 0.62 | 0.56 |
| 15 | 0.45 | 0.54 |
| 16 | 0.49 | 0.51 |
| 17 | 0.25 | 0.35 |
| 18 | 0.39 | 0.38 |
| 19 | 0.57 | 0.71 |
| 20 | 0.43 | 0.53 |
| 21 | 0.23 | 0.31 |
| 22 | 0.19 | 0.25 |
| 23 | 0.17 | 0.15 |
| 24 | 0.24 | 0.19 |
| 25 | 0.22 | 0.32 |
| 26 | 0.31 | 0.29 |
| 27 | 0.43 | 0.51 |
| 28 | 0.58 | 0.52 |
| 29 | 0.66 | 0.69 |
| 30 | 0.62 | 0.73 |
| 31 | 0.73 | 0.83 |
| 32 | 0.75 | 0.75 |
| 33 | 0.54 | 0.63 |
| 34 | 0.26 | 0.31 |
| 35 | 0.29 | 0.36 |
| 36 | 0.38 | 0.39 |
| 37 | 0.61 | 0.66 |
| 38 | 0.16 | 0.21 |
| **Correlation ratio** | **0.946902916** |  |
| **The inv(16)/t(16;16) AML patients with SOCS1 down- regulation caused by ubiquitin degradation** | **The decreased protein levels of CUEDC2 in inv(16)/t(16;16) AML primary cells** | **The decreased protein levels of SOCS1 in inv(16)/t(16;16) AML primary cells** |
| 1 | 0.38 | 0.49 |
| 2 | 0.26 | 0.28 |
| 3 | 0.27 | 0.35 |
| 4 | 0.39 | 0.45 |
| 5 | 0.21 | 0.26 |
| 6 | 0.28 | 0.34 |
| 7 | 0.55 | 0.48 |
| 8 | 0.39 | 0.35 |
| 9 | 0.33 | 0.39 |
| 10 | 0.42 | 0.38 |
| 11 | 0.47 | 0.56 |
| 12 | 0.48 | 0.43 |
| 13 | 0.65 | 0.61 |
| 14 | 0.53 | 0.65 |
| 15 | 0.66 | 0.73 |
| 16 | 0.22 | 0.23 |
| 17 | 0.24 | 0.26 |
| 18 | 0.37 | 0.36 |
| 19 | 0.34 | 0.36 |
| 20 | 0.45 | 0.48 |
| 21 | 0.58 | 0.66 |
| 22 | 0.61 | 0.72 |
| 23 | 0.46 | 0.56 |
| 24 | 0.67 | 0.59 |
| 25 | 0.77 | 0.88 |
| 26 | 0.72 | 0.82 |
| 27 | 0.19 | 0.25 |
| 28 | 0.36 | 0.44 |
| 29 | 0.68 | 0.78 |
| 30 | 0.44 | 0.59 |
| 31 | 0.32 | 0.34 |
| 32 | 0.51 | 0.61 |
| 33 | 0.15 | 0.26 |
| 34 | 0.55 | 0.65 |
| 35 | 0.62 | 0.65 |
| 36 | 0.71 | 0.73 |
| 37 | 0.18 | 0.22 |
| 38 | 0.64 | 0.72 |
| 39 | 0.69 | 0.75 |
| 40 | 0.31 | 0.41 |
| 41 | 0.7 | 0.81 |
| **Correlation ratio** | **0.953990557** |  |
