# Supplementary material for: CUEDC2, a novel interacting partner of the SOCS1 protein, plays important roles in the leukaemogenesis of acute myeloid leukaemia
Source: Cell Death Dis. 2018 Jul 10;9(7):774. doi: 10.1038/s41419-018-0812-6 (PMC6039501; doi:10.1038/s41419-018-0812-6)
Supplement: Supplementary file 4 — Table S3 [file 41419_2018_812_MOESM4_ESM.doc]

Table S3. The Nucleotide Sequences of CUEDC2 shRNA Scrambled RNA and primers for pWPXLd-CUEDC2.

| Names | Nucleotide Sequences |
| --- | --- |
| CUEDC2 ShRNA-1 | 5’-GCTCCATAGTGTTAACCTACT-3’ |
| CUEDC2 ShRNA-2 | 5’-GCCCGAAATGCTCAAAGAAGA-3’ |
| Scrambled RNA | 5’-GATGCCCGAACAAGTCAAGAA-3’ |
| CUEDC2-F | 5’-CATGGATCCGAGAGCATGGAGCTG-3’ |
| CUEDC2-R | 5’-GCTACGCGTTGATGGAAGCGGTA CT-3’ |
