# Supplementary material for: CUEDC2, a novel interacting partner of the SOCS1 protein, plays important roles in the leukaemogenesis of acute myeloid leukaemia
Source: Cell Death Dis. 2018 Jul 10;9(7):774. doi: 10.1038/s41419-018-0812-6 (PMC6039501; doi:10.1038/s41419-018-0812-6)
Supplement: Supplementary file 5 — Summary and Figure legend for supplemnbtary date [file 41419_2018_812_MOESM5_ESM.doc]

**Summary:**

The most possible interaction protein of SOCS1 have been summarized **in Table S1**, the accession number, gene name, protein name, unique peptides detected and coverage were all listed. The correlation ratios of the decreased protein levels between CUEDC2 and SOCS1 in the AML cell lines and different AML subtype primary cells with SOCS1 downregulation caused by ubiquitin degradation were analyzed by the Pearson correlation method, and the result was summarized **in Table S2.** Our results indicated that the decreased protein levels of CUEDC2 had a positive correlation with the decreased protein levels of SOCS1 caused by ubiquitin degradation. The Nucleotide Sequences of CUEDC2 shRNA Scrambled RNA and primers for pWPXLd-CUEDC2 was summarized **in Table S3**. And the correlation between the expression of CUEDC2 and overall survival (OS) and event-free survival (EFS) of AML patients was also investigated and summarized as **Figure S1**, the low expression predict a low OS and EFS of AML patients.

**Figure S1 Effects of the expression of CUEDC2 on the overall survival (OS) and event-free survival (EFS) of AML patients.** (a) The low expression of CUEDC2 in AML patients might indicate a low OS (n=188, P=0.0018). (b) The low expression of CUEDC2 in AML patients might indicate a low EFS (n=188, P=0.0014).
